# Jejunal diverticulosis complicated with perforation: A rare acute abdomen etiology

**DOI:** 10.1016/j.ijscr.2019.09.013

**Published:** 2019-09-23

**Authors:** Ahmet Gökhan Sarıtaş, Uğur Topal, İsmail Cem Eray, Kubilay Dalcı, Atılgan Tolga Akçamı, Kıvılcım Erdoğan

**Affiliations:** a Department of General Surgery, Cukurova University Faculty of Medicine, 01330, Cukurova, Adana, Turkey; b Department of Pathology, Cukurova University Faculty of Medicine, 01330, Cukurova, Adana, Turkey

**Keywords:** Jejunal, Diverticular, Perforation

## Abstract

**Introduction:**

Jejunal diverticulosis is a rare intestinal pathology with an incidence of 0.5–1%. While most cases are asymptomatic, 30–40% of the cases may become symptomatic with chronic abdominal pain, malabsorption, hemorrhage, diverticulitis, obstruction, abscess formation and, rarely, diverticula perforation. It is generally localized on the mesenteric side and it develops from the entry points of the vessels into the jejunum.

**Case presentation:**

Case 1 – A 36-year Case 2 a 75 old female patient patient was admitted to the emergency department with the complaint of widespread abdominal pain, Case 1 8 diverticules, one with diverticule perforation, was observed in the jejunum segment between the 50th and 90th centimeters after the Treitz Ligament. Case 2 – In the diverticulum 100 cm distal from the Treitz ligament, mesenteric perforation area of 4–5 mm were observed.

**Results:**

Segmentary small bowel resection and side-by-side anastomosis were performed in these cases. In the histopathological examination, the Diverticula were seen to be pseudodiverticullar lesions including herniation of the mucosa and submucosa.

**Conclusion:**

Jejunoileal diverticulosis is a rare disease with life-threatening complications such as perforation, obstruction and bleeding, it is usually asymptomatic or presents with nonspecific symptoms. It should be considered in the differential diagnosis of acute abdomen.

## Highlights

• Physicians treating this heterogeneous disease need to know the complex underlying mechanisms as well as the multiple management options.
• Operative approach is still the definitive treatment and can be preferred to improve patients’ quality of life and to prevent more severe symptoms from developing.
• Rare and difficult diagnosis of jejunal diverticulum perforation in elderly patients presenting with acute abdomen should be considered in the differential diagnosis.

## 1 Introduction – objective

Jejunal diverticulosis is a rare intestinal pathology with an incidence of 0.5–1% [1]. In the etiology, the pressure increase in the small intestinal lumen and the weakening of the wall is blamed [2].

While most cases are asymptomatic, 30–40% of the cases may become symptomatic with chronic abdominal pain, malabsorption, hemorrhage, diverticulitis, obstruction, abscess formation and, rarely, diverticula perforation [[2], [3], [4]].

In this article, we aimed to present a case of jejunal diverticulosis and diverticulum perforation which is a rare etiology in the presentation of acute abdominal pain. This work is reported in line with the Preferred Reporting of Case Series in Surgery (PROCESS) Guidelines criteria [5].

## 2 Case – 1

**Fig. 1:**
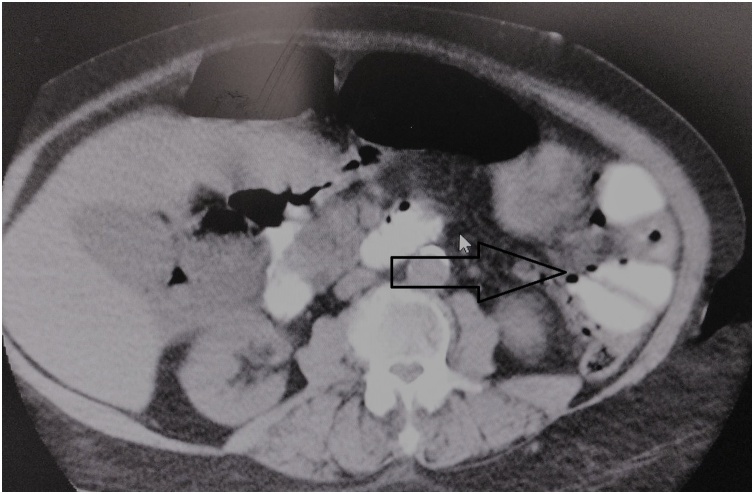
Free fluid compatible and edema in the mesentery the small bowel loops in the left upper quadrant.

**Fig. 2:**
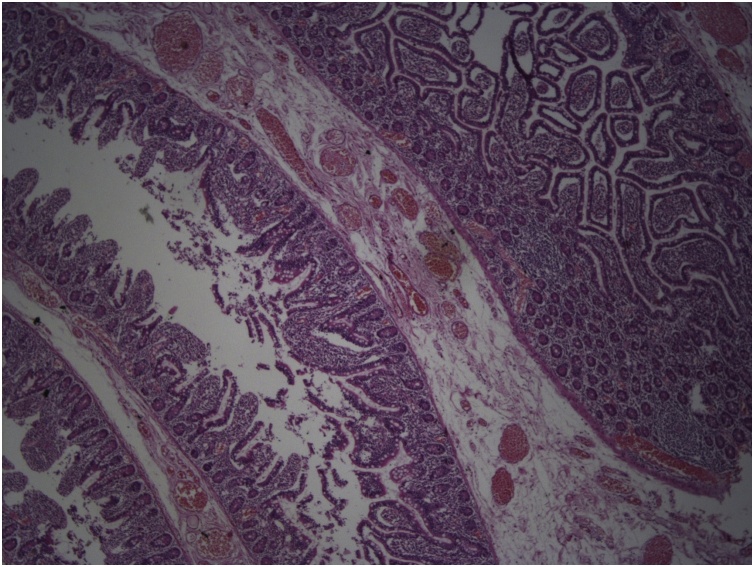
H&E X40 Diverticulare were seen to be pseudodiverticullary lesions including herniation of the mucosa and submucosa. In the histopathological examination.

A 36-year-old female patient was admitted to the emergency department with the complaint of widespread abdominal pain, nausea and vomiting. Physical examination revealed widespread defense, rebound and sensitivity in all quadrants. Temp: 37.6’C, Pulse: 114/min, TA: 110/80 mmHg, WBC: 15,3. In the patient, who was operated on for endometriosis 15 days prior to her complaints, direct abdominal X-ray was performed for determining acute abdomen etiology and it revealed free air under the diaphragm. Computed tomography (CT) imaging performed to clarify the cause completely revealed free air around the liver, free fluid compatible with intense collection, and edema in the mesentery (Fig. 1). The patient was operated under emergency conditions and diffuse purulent fluid with bile, 8 diverticuli, one with diverticula perforation, was observed in the jejunum segment between the 50th and 90th centimeters after the Treitz Ligament. Segmentary small bowel resection, side-by-side anastomosis and intraabdominal irrigation were performed. The patient was discharged from the hospital on the 7th postoperative day with full recovery. In the pathology of the specimen, 8 diverticuli, the largest one being 8 × 7 cm, and one with a 4 mm perforation area were detected. Diverticula were seen to be pseudodiverticullar lesions including herniation of the mucosa and submucosa. In the histopathological examination (Fig. 2), a diverticulum area lined with normal intestinal epithelium and showing continuity in submucosal and submucosal muscular layers were observed. The patient had no specific postoperative complications and no wound complication developed. We did not need re-discovery/revision surgeries. We did not experience post-operative 30-day and long-term morbidity/mortality.

## 3 Case – 2

**Fig. 3:**
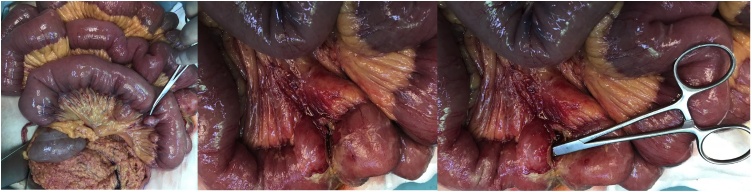
A large number of diverticula cysts with mesenteric origin.

A 75-year-old female patient who had complaints of abdominal pain, nausea, vomiting and fever for two days was admitted to the emergency clinic. In the physical examination TA: 140/80 mmHg, Pulse: 120/min, Respiratory rate: 30/min, Temp: 37.8 °C was observed; widespread sensitivity, rebound and defense was present during the abdominal exam. There was no pathology in the rectal exam. Laboratory values were WBC: 7.100 mm^3^, Hgb: 11.1 g/dl, Plt: 174.000. She had a history of Atrial Fibrillation and Heart Failure. On posterior anterior (PA) chest radiography, free air under the diaphragm was not seen. Abdominal CT imaging showed edema in the small bowel loops in the left upper quadrant. The patient was operated with the diagnosis of acute abdomen. From the 70th to 120thcm from the Treitz ligament, a large number of diverticular cysts with mesenteric origin were observed (Fig. 3). In the diverticulum 100 cm distal from the Treitz ligament, inflammation, edema and mesenteric perforation area of 4–5 mm were observed. Segmentary small bowel resection and side-by-side anastomosis were performed. Patient was discharged with full recovery. The patient had no specific postoperative complications any wound complication did not develop. We did not need re-discovery/revision surgeries. We did not experience post-operative 30-day and long-term morbidity/mortality.

## 4 Discussion

Acquired jejunoileal diverticulosis was first described by Sommering in 1794 and later by Sir Astley Cooper in 1807 as herniation of the mucosa and the submucosa of the mesenteric side of the small intestinal wall along the muscular layer (pseudodiverticule) (6–7). Non-Meckel small bowel diverticular disease has a prevalence of 0.3–1.3% in postmortem studies, 0.5–1.9% in contrast-enhanced imaging modalities, and is more common in older patients [8,9].

Duodenum is the most common localization of small bowel diverticular disease; the incidence of jejunum and ileum is less, with 0.7–1% [[8], [9], [10], [11]]. The incidence of diverticula in the colon simultaneously with the jejunoileal diverticulum is 20–70%, in the duodenum 10–40%, in the esophagus and stomach 2% [[8], [9], [10], [11], [12]].

The etiology of jejunal diverticulosis is unknown and is thought to be caused by a combination of abnormal peristalsis, intestinal dyskinesia and increased intraluminal pressure factors. It is generally localized on the mesenteric side and it develops from the jejunal entry points of the vessels [[4], [5], [6], [7], [8], [9], [10], [11], [12]].

Uncomplicated cases are usually asymptomatic. It may show symptoms such as nausea, vomiting, epigastric and periumbilical abdominal pain. Complications such as diverticulitis, bleeding, intestinal obstruction and perforation occur in 30% of patients with jejunal diverticulosis [[2], [3], [4], [5], [6], [7], [8], [9], [10], [11], [12]]. Lobo et al. described the most frequent complication requiring surgery in small intestine diverticulosis as perforation [13].

Jejunal diverticulosis gives a bullion-like appearance on the barium x-ray [6,[14], [15], [16]]. Although enteroclysis and enterography are the best imaging modalities in diagnosis, their use in emergency situations is limited [17]. CT imaging is the best imaging method for diagnosing complicated jejunal diverticulosis. The "whirlpool" sign on abdominal CT defines the spiral, sharp-appearing mesenteric vessels feeding the intestinal loops [18].

Most of the complications of jejunoileal diverticulosis requires surgical treatment. Surgical treatment of excision of simple diverticula is not recommended because of the risk of postoperative intestinal leak, sepsis and death; intestinal resection and anastomosis should be preferred [6]. In the presented case, segmental small intestinal resection and side-by-side anastomosis with staplers were performed due to diverticulum perforation.

In the differential diagnosis of jejunal diverticulosis perforation, Crohn's disease, small intestinal neoplasm, foreign body trauma, and colonic diverticulitis perforation should be considered [7,19].

Jejunoileal diverticulosis is a rare disease with life-threatening complications such as perforation, obstruction and bleeding, usually with asymptomatic or nonspecific symptoms. It should be considered in the differential diagnosis of abdominal pain and acute abdomen.

## Funding

We have no supportive funding.

## Ethical approval

I certify that this kind of manuscript does not require ethical approval.

## Consent

Written informed consent for publication of his clinical details and clinical images was obtained from the patient.

## Author contribution

Uğut topal.-Ahmet gökhan sarıtaş study concept, writing the paper, final decision to publish, data collection.

İsmail cem eray, Kıvılcım erdoğan and Kubilay dalcı. - study concept, data collection.

Atılgan tolga akçam İsmail cem eray,Kıvılcım erdoğan and Kubilay dalcı. - data collection and analysis.

## Registration of research studies

We registered our study with the Research Registry. Our unique identifying number is: research registry 5107.

## Guarantor

Ahmet Gökhan Sarıtaş, İsmail Cem Eray.

## Provenance and peer review

Not commissioned, externally peer-reviewed

## Declaration of Competing Interest

No conflicts of interest were declared.
